# Frontotemporal EEG as potential biomarker for early MCI: a case–control study

**DOI:** 10.1186/s12888-022-03932-0

**Published:** 2022-04-22

**Authors:** Yasue Mitsukura, Brian Sumali, Hideto Watanabe, Toshiharu Ikaga, Toshihiko Nishimura

**Affiliations:** 1 Department of System Design Engineering, School of Integrated Design Engineering, Faculty of Science and Technology, Keio University, Yokohama, Kanagawa Japan; 2 Keio Global Institute(KGRI), Keio University, Tokyo, Japan; 3 Department of Anesthesia, School of Medicine, Stanford University, Stanford, CA USA

**Keywords:** Dementia, MCI, EEG, Clinical decision-making

## Abstract

**Background:**

Previous studies using EEG (electroencephalography) as biomarker for dementia have attempted to research, but results have been inconsistent. Most of the studies have extremely small number of samples (average *N* = 15) and studies with large number of data do not have control group. We identified EEG features that may be biomarkers for dementia with 120 subjects (dementia 10, MCI 33, against control 77).

**Methods:**

We recorded EEG from 120 patients with dementia as they stayed in relaxed state using a single-channel EEG device while conducting real-time noise reduction and compared them to healthy subjects. Differences in EEG between patients and controls, as well as differences in patients’ severity, were examined using the ratio of power spectrum at each frequency.

**Results:**

In comparing healthy controls and dementia patients, significant power spectrum differences were observed at 3 Hz, 4 Hz, and 10 Hz and higher frequencies. In patient group, differences in the power spectrum were observed between asymptomatic patients and healthy individuals, and between patients of each respective severity level and healthy individuals.

**Conclusions:**

A study with a larger sample size should be conducted to gauge reproducibility, but the results implied the effectiveness of EEG in clinical practice as a biomarker of MCI (mild cognitive impairment) and/or dementia.

## Background

The objective of this study was to propose a novel dementia diagnosis system and biomarkers for early MCI (mild cognitive impairment) detection and dementia by utilizing a simple electroencephalography (EEG) device.

Dementia has become a public issue in Japan. It is said that one in seven elderly people of 65 years old or more is affected with dementia, and it was predicted that the total dementia patients in Japan will reach around 6.5 million to 7 million people in 2025 [1]. Dementia is different from other illness in that it requires both public and family care. In total, the cost for the dementia care is estimated to be approximately 14.5 trillion Japanese Yen (JPY) per year and it is very taxing for the Japanese society. As a comparison, cancer is estimated to cost around 9.7 trillion JPY per year [2].

Recently, research projects are focused on prevention and early detection of dementia. These are especially important because there is no cure or treatment for dementia yet. Popular methodologies for early dementia detection utilize neuroimaging techniques such as Magnetic Resonance Imaging (MRI) and Positron Emission Tomography (PET). Although they are accurate, neuroimaging techniques are both expensive and time consuming. Additionally, conventional neuroimaging techniques can only detect dementia when it has been sufficiently developed – i.e., the patient’s brain has suffered irreversible structural damage. As a result, the usage of EEG as simpler, cheaper, and easier alternative to neuroimaging is starting to become popular.

EEG features has been successfully used to detect Huntington disease and epilepsy, proving their reliability. Moreover, as EEG reflects functional changes in the cerebral cortex, detecting dementia before permanent structural damage in brain might be possible using EEG [3]. In addition to dementia, Mild Cognitive Impairment (MCI) detection is also an important task. MCI is said to be the early stages of dementia, and MCI-afflicted patients are more likely to progress into dementia, compared to healthy people.

Most of conventional studies that utilized EEG for dementia diagnosis system used multi-channel EEG devices and did not consider the diagnosis of MCI [4]. Additionally, previous studies related to EEG biomarkers suffered from low number of samples of lack of control groups. Moreover, one of the conventional studies that utilize EEG to assess MCI and AD is from Meghdadi et al. [5], which successfully built a machine learning for dementia / MCI diagnosis system using multi-channel EEG setup; unfortunately, multichannel EEG device is hard to wear and burdensome to patients. Here, this study aims to propose a novel dementia-MCI diagnosis system and the possibility of utilizing a comfortable, single-channel EEG device as biomarkers for clinical screening.

As the proposed system utilized a single-channel EEG device, it required less preparation time and also less burdensome to the experimental subjects; it can be said that the proposed system was an improvement from the conventional systems. Another objective of this research was to verify the validity of the dementia diagnosis system using the simple measurement by evaluating the feature values using statistical analysis and machine learning.

## Methods

### Participants

The participants of this study were 120 people (67% female) aged 40 years old up to 91 years old. The study was conducted from year 2016 to 2019. The mean age of the participants was 67.0 ± 9.19 years old. EEG, MRI (in particular, FA-BHQ and GM-BHQ values were obtained), and cognitive screening tests were conducted to screen the participants. However only MMSE is utilized in this study as variable. This study was approved by the ethics committees of Keio University and performed in accordance with the Declaration of Helsinki. Written informed consent was obtained from all participants or their legal guardian. This study is a case–control study and is observational in nature; all obtained data were made available to the caregiver and / or the participant. The study protocol was approved by the Keio University Ethics Review Board with approval no.: 28–20, 28–59, 29–33, 30–96, 31–56.

Exclusion criteria for patients were: 1) persons who have physical or psychiatric disorders that impede the use of EEG; 2) persons who have comorbid psychiatric disorders other than dementia; 3) persons who have comorbidities that could interfere with EEG recordings, such as brain tumors, stroke, or epilepsy.

For the comparison, reference data for healthy individuals that were obtained separately from this study were used. Inclusion criteria for healthy individuals were: 1) no history of mental illness; 2) legal adult defined by Japanese law (age ≥ 20 years). The healthy volunteer’s age is matched as closely as possible to the dementia patients. It was also required that they do not meet the exclusion criteria for dementia patients listed above.

All participants were Japanese (Asian) and were divided into three groups: dementia (*N* = 10), MCI (*N* = 33), and control (*N* = 77). From all of the participants, 7 were diabetic, 7 were obese, 24 had hyperlipidemia, 4 were diagnosed as clinically depressed, 3 had history of neurological diseases, 35 had hypertension, 2 had history of stroke, 1 had history of myocardial infarction, 14 had history of allergic rhinitis, 1 had history of COPD, 4 had asthma, 12 had skin condition, 9 had arthritis, 20 had low back pain, 54 had osteoporosis, 4 had history of kidney problem, and 2 had history of cancer. Dementia patients and MCI patients in this study were all Alzheimer-type.

#### EEG acquisition

A Participants were asked to wear a single channel EEG device (NeuroSky Single Channel EEG, Original noise reduction BMD version). EEG was taken during closed-eye relaxed state for a total of 100 s. The location of the electrode was Fp1 according to the 10–20 international system (left prefrontal region) and the measurement device was MindWave Mobile II BMD II ver. with sampling rate of 512 Hz. Mini-mental state examination (MMSE) was utilized as the cognitive screening test. MMSE is a 30-points question which is commonly used for assessing dementia. Subjects who scored 24 points or lower were labeled as dementia while patients with score of 25 – 27 is labeled as MCI. Subjects with score of 28 or higher is labelled as healthy.

### Analysis

#### Data preprocessing

For the EEG device used in this study, independent verification has already demonstrated that the device can reliably remove environmental noise and unintended frequencies [6]. Signals acquired from Fp1 using a monopole EEG were passed through a bandpass filter of 1–30 Hz to extract EEG components [7]. However, even if non-target frequencies can be removed, the acquired data come with noise caused by muscle movement or blinking. To remove these noises, a filter created for this purpose was used. This filter acquires the patterns of body movement and blinking in advance, and the threshold value is automatically set according to the situation. We adopted conventional methods as noise reduction [6, 8, 9] This procedure reduces computation costs and removes blinks, body movements, and electrical noise in real time.

In order to account for individual differences in EEG amplitude, normalization was performed with an average of 0 and a dispersion of 1 for the filtered signal. Subsequently, a fast Fourier transform was performed to calculate the power spectrum. Also, in order to clarify the difference between healthy individuals and each patient group with dementia and MCI, the average power spectrum of the comparison target group was set as 1; i.e., a relative power spectrum value was used.

The sampling interval of the EEG device was set to 512 Hz. Therefore, the amount of data for each individual was 51,200 samples of 100 × 512. Each individual’s EEG data were translated to the frequency domain by Fourier transform per second.

#### Statistical analysis

First, all acquired data were divided into three groups according to their MMSE score: patients with dementia, patients with MCI, and healthy controls. Descriptive statistics were used to describe the study participants. Distributions of all variables were inspected using histograms, q-q plots, and Shapiro-Wilks tests before conducting statistical analyses. Statistical significance was set at two-tailed *p* < 0.05, and we used false discovery rate (FDR) to control for multiple comparisons. Demographic variables for patients with dementia, MCI, and healthy individuals were compared by two-sample t test and/or chi-square test. For the EEG comparisons, patients’ EEG power spectra are expressed as a ratio when the average of the power spectra of healthy individuals is 1, as mentioned above.

#### Signal processing

As a preprocessing, noise removal was performed to the obtained EEG data and then transformed to the frequency-domain. The noise removal was performed using Summation of Derivatives within Windows (SDW) algorithm and Ensemble Empirical Mode Decomposition (EEMD).

The SDW method detects noise by using the sum of first derivatives within a window [10]. The window selected in this study is 2 s, according to the conventional methods, shown in Table 1. The signal was then decomposed into several intrinsic mode functions (IMFs) by applying EEMD in the interval detected by the SDW method.

**Table 1:** Conventional EEG studies using 2 s window

Reason	Papers
Unspecified	[11–18]
Utilized several power spectra with different window lengths	[19]
Artifact removal	[20–24]
LORETA analysis	[25–27]
The duration of EEG is 2 to 4 s	[28, 29]

The components with a mean cross-correlation function between IMFs greater than 0.5 were defined as noise and removed. Remaining components were then summed up to reconstruct the clean signal. For frequency transformation, the short-time Fourier transform (STFT) was used. The frequency features were the frequency bins 1-45 Hz, averaged between the windows, and then normalized. The average of the power spectra for each EEG band was also calculated and used as the frequency features. The specification of EEG bands were as follows: Delta (δ): 1–4 Hz, Theta (θ) 4–8 Hz, Alpha (α) 8–13 Hz, Alpha-1 (α1) 8–9 Hz, Alpha-2 (α2) 9–11 Hz, Alpha-3 (α3) 11–13 Hz, Beta (β) 13–30 Hz, Beta-1 (β1) 13–20 Hz, Beta-2 (β2) 20–30 Hz, and Gamma (γ) 30–45 Hz.

Next, the subjects were labelled according to their MMSE score: dementia, MCI, and healthy. As there was imbalance in the number of samples, Synthetic Minority Over-sampling Technique (SMOTE) algorithm was applied to artificially create new samples [30].

In order to verify whether the frequency features used were effective in discriminating between classes, a significant difference test between classes was conducted. First, a nonparametric test, Kruska-wallis test was performed. Then, multiple comparison with Bonferroni correction was performed. The significance level was set as 5%.

Next, the statistically significant features were utilized as predictors for Support Vector Machines (SVM) to solve the classification problem. Although SVM is known to be able to handle linearly separable data, by utilizing kernel trick and computing the maximum margin, it can sufficiently handle nonlinear data.

In this study, SVM with radial basis function kernel was utilized as the classifier. In order to evaluate the classification performance, tenfold cross-validation was performed.

## Results

### Feature selection results

**Fig. 1:**
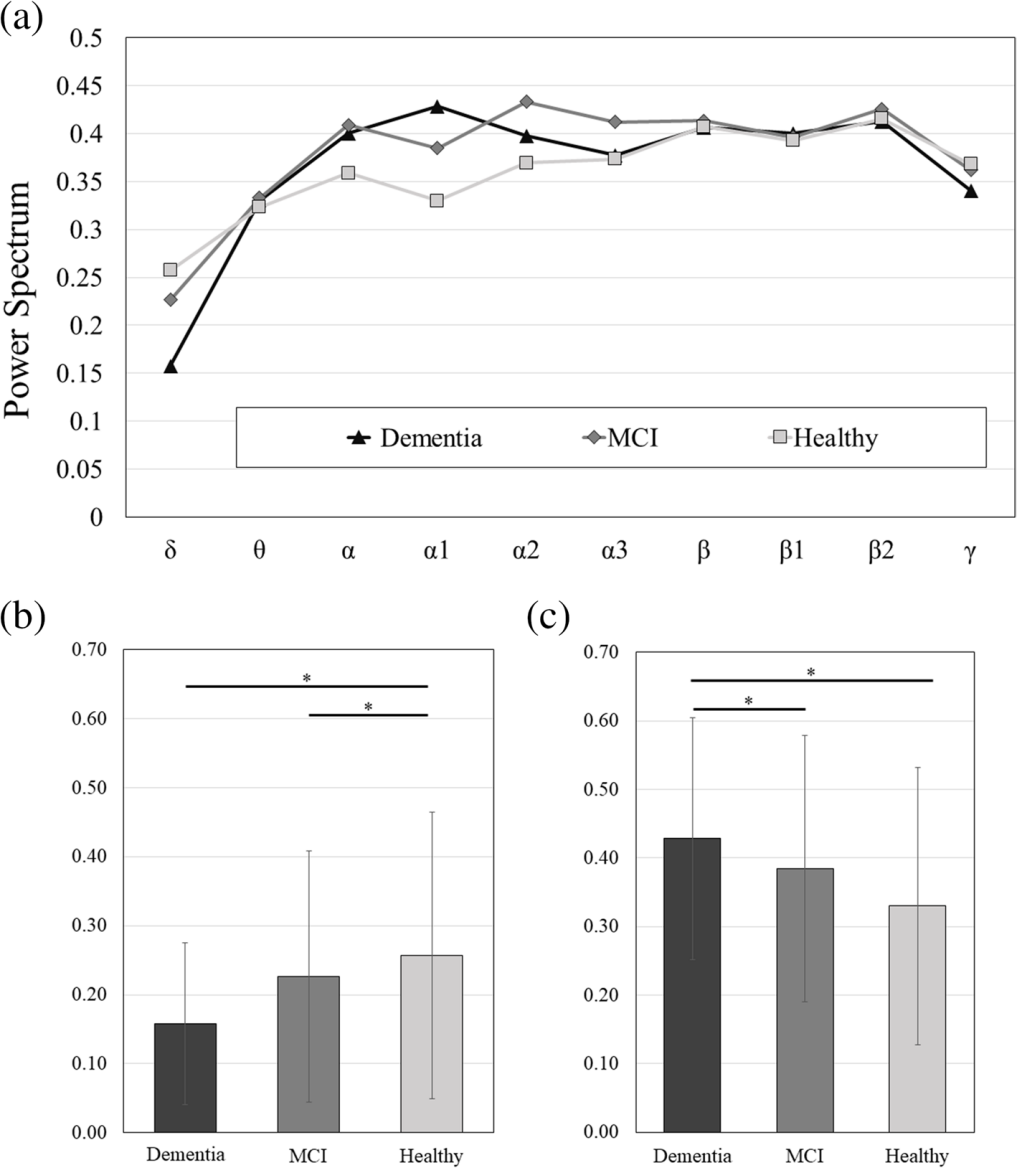
**a** Comparison of the power spectra of each EEG band; **b c** Comparison of power spectra in δ and α1 EEG bands which have significant differences, respectively

The power spectra comparison for each band was shown in Fig. 1a. The significance testing for the three groups showed significantly increased delta band for the healthy group compared to dementia group and MCI group, as shown in Fig. 1b, and significantly increased alpha-1 band for the dementia group compared to MCI group and healthy group as shown in Fig. 1c.

### Results of accuracies

The classification results for the three classes of dementia group, MCI group, and healthy group are shown in Table 2. The classification accuracy rate, sensitivity, and specificity for the training data were all 100%, while the accuracy rate, sensitivity, and specificity for the training data were 86.32%, 78.91%, and 85.36%, respectively.

**Table 2:** Classification results

	Training	Testing
Accuracy (%)	100.0	86.32
Sensitivity (%)	100.0	78.91
Specificity (%)	100.0	85.36

## Discussion

### Feature selection results

The findings were in line with conventional studies of higher power in low-frequencies range for healthy subjects and higher power in high-frequencies range for dementia and MCI patients [31]. Many of frequency bins also showed significant differences between the classes and were chosen as predictors. These predictors might also be utilized as EEG biomarkers for dementia and MCI in clinical scene.

The statistical analysis result showed that the power in the low-frequency region of the dementia and MCI patients was higher than that of the healthy subjects, and that the power in the high-frequency region was lower, similar to conventional studies which utilized multi-channel EEG device.

### Results of accuracies

It was confirmed that the power spectra of the frequency bands and frequency bins selected as features were sufficient to discriminate among the three classes of dementia, MCI, and normal. These results were comparable in accuracy to previous studies using multichannel EEG device with accuracy of 88.89% [32]. From these results, it was shown that it is possible to obtain a new biomarker for dementia and early MCI by using a simple EEG device that acquires only single channel.

## Conclusion

The objective of this study was to develop a system for diagnosing dementia and early detection of dementia using only EEG. The experimental subjects were divided into three classes according to their MMSE scores: dementia group, MCI group, and healthy group. Feature extraction was performed, and the validity of the features were verified by constructing a machine learning model that can differentiate and label the subjects into the three classes.

The statistical analysis result showed that the power in the low-frequency region of the dementia and MCI patients was higher than that of the healthy subjects, and that the power in the high-frequency region was lower, similar to conventional studies which utilized multi-channel EEG device.

Features other than EEG frequencies and different classifiers might be considered as a future work to improve the classification accuracy.

## Data Availability

The datasets generated and/or analysed during the current study are not publicly available due to restrictions from IRB (identifying and potentially sensitive information) but are available from the corresponding author on reasonable request.

## Abbreviations

EEG: Electroencephalography
EEMD: Ensemble Empirical Mode Decomposition
IMFs: Intrinsic Mode Functions
JPY: Japanese Yen
MCI: Mild Cognitive Impairment
MMSE: Mini-mental state examination
MRI: Magnetic Resonance Imaging
PET: Positron Emission Tomography
SDW: Summation of Derivatives within Windows
SMOTE: Synthetic Minority Over-sampling Technique
STFT: Short-time Fourier transform
SVM: Support Vector Machines
